# Potential Use of Waste Activated Sludge Hydrothermally Treated as a Renewable Fuel or Activated Carbon Precursor

**DOI:** 10.3390/molecules25153534

**Published:** 2020-08-02

**Authors:** J. A. Villamil, E. Diaz, M. A. de la Rubia, A. F. Mohedano

**Affiliations:** 1 Departamento de Tecnología Química y Ambiental, Universidad Rey Juan Carlos, 28933 Móstoles (Madrid), Spain; 2 Departamento de Ingenieria Química, Facultad de Ciencias, Universidad Autonoma de Madrid, Campus de Cantoblanco, 28049 Madrid, Spain; elena.diaz@uam.es (E.D.); angeles.delarubia@uam.es (M.A.d.l.R.); angelf.mohedano@uam.es (A.F.M.)

**Keywords:** activated carbon, adsorption, chemical activation, hydrothermal carbonization, hydrochar, low-cost adsorbent, physical activation

## Abstract

In this work, dewatered waste activated sludge (DWAS) was subjected to hydrothermal carbonization to obtain hydrochars that can be used as renewable solid fuels or activated carbon precursors. A central composite rotatable design was used to analyze the effect of temperature (140–220 °C) and reaction time (0.5–4 h) on the physicochemical properties of the products. The hydrochars exhibited increased heating values (up to 22.3 MJ/kg) and their air-activation provided carbons with a low BET area (100 m^2^/g). By contrast, chemical activation with K_2_CO_3_, KOH, FeCl_3_ and ZnCl_2_ gave carbons with a well-developed porous network (BET areas of 410–1030 m^2^/g) and substantial contents in mesopores (0.079–0.271 cm^3^/g) and micropores (0.136–0.398 cm^3^/g). The chemically activated carbons had a fairly good potential to adsorb emerging pollutants such as sulfamethoxazole, antipyrine and desipramine from the liquid phase. This was especially the case with KOH-activated hydrochars, which exhibited a maximum adsorption capacity of 412, 198 and 146 mg/g, respectively, for the previous pollutants.

## 1. Introduction

The current massive production of sewage sludge by wastewater treatment plants (WWTP) has raised the need for new, more effective solutions for managing this waste. Spain alone produces around 1.4 million tons (d.b.; dry basis) of biosolids each year and the EU countries, in combination, generate more than 10.5 million tons. Such biosolids are mainly used for agricultural (70%) or landfilling purposes (14%) [1]. Various techniques including combustion [2], gasification [3,4], pyrolysis and activation [5,6] have been used for the thermal valorization of biosolids in the past two decades. These techniques, however, use large amounts of energy to dry the raw material, require strict control of emitted pollutants, and in many cases, elicit social and political rejection [7,8].

Hydrothermal carbonization (HTC), also referred to as “wet torrefaction”, is an exothermic process that allows biomass to be converted at mild temperatures (170–250 °C) and short residence times (5–240 min) by using autogenous pressure and water as reaction medium. As it requires no previous drying of the raw material, HTC uses energy sparingly, so it is cost-effective and environmental friendly. The main reaction product from the thermal treatment of biomass is a carbonaceous solid (hydrochar) formed by hydrolysis, decarbonylation, decarboxylation, dehydration, polymerization and condensation reactions [9]. Hydrochar can be directly used as a solid fuel similar to sub-bituminous coal [10], or alternatively, for soil remediation [11], CO_2_ sequestration [12], catalysis [13] and adsorption purposes [12]. The hydrothermal treatment additionally produces a gas phase and processes water (PW). The gaseous stream mainly contains CO_2_ (>90%) and small amounts of CH_4_, H_2_ and CO [14,15]. PW contains up to 15% of the initial carbon present in the sludge, in addition to large amounts of organic compounds such as volatile fatty acids (acetic and propionic), carbohydrates, aldehydes, furans, phenols, pyrazines and pyrroles [10,16,17]. As it contains substantial amounts of macro (N and P) and micronutrients (Al, Ca, Fe, Mg), PW could be directly used as fertilizer [18,19]. Additionally, organic matter present in PW can be subjected to oxidation in wet air [20] or valorized by anaerobic digestion to obtain methane [16,17,21,22,23,24,25,26].

The valorization of sewage sludge by HTC is arousing increasing interest on account of the potential of the resulting hydrochar (HC) for use in various fields (mainly as solid fuel provided that it has an appropriate composition) [27,28]. However, hydrochars with high nitrogen and sulfur contents can produce NO*_x_* and SO*_x_* gases during combustion, or leave large amounts of residual ash that can cause reactor slagging and corrosion [29], thereby decreasing the efficiency of the thermochemical process [30]. Some authors have examined the effects of temperature, reaction time and solid loading on various characteristics of hydrochars (particularly their energy content and physicochemical properties) [31,32,33,34,35]. Combustion of hydrochars from sewage sludge can be improved through cohydrothermal carbonization (viz., by blending the sludge with biomass from sawdust, cornstalks or pig manure, for example, before heating) [36,37,38,39] or mixing with another fuel such as coal [40]. Further, adding an acid to the sludge has been found to increase the energy content of the resulting hydrochar up to 28.5 MJ/kg [37].

While hydrochars only contain few pores, their surface area can be increased by physical and chemical activation [29]. This has rarely been the case with activated sewage sludge, however. Saeta et al., [41] examined adsorption of the dye Methyl Blue on a sewage sludge hydrochar obtained by heating at 200 °C for 1 h, which was subsequently steam-activated at 900 °C for 30, 60 or 120 min. The resulting activated hydrochars exhibited a large surface area (483–595 m^2^/g) and a high adsorption capacity (134–162 mg/g). Spataru et al. [42] succeeded in removing up to 97% of orthophosphate from wastewater by adsorption in a hydrochar obtained by HTC at 210 °C for 5 h, which was immediately activated with KOH.

In this work, we assessed the potential of HTC to valorize DWAS in the form of hydrochars of potential use as solid fuels, soil amendments and activated carbon precursors. For this purpose, the influence of temperature and the reaction time in the HTC process was examined by using a central composite rotatable design. The resulting hydrochars were subjected to physical activation with air at different temperatures (300–450 °C) and also to chemical activation with K_2_CO_3_, KOH, FeCl_3_ or ZnCl_2_ at 650 or 850 °C. The activated materials were used as adsorbents for the pharmaceuticals sulfamethoxazole, antipyrine and desipramine in an aqueous phase to assess the influence of p*K*_a_ on the adsorption capacity of the solids.

## 2. Methods

### 2.1. Dewatered Waste Activated Sludge

DWAS was collected from a full-scale membrane bioreactor used to treat cosmetic wastewater in Madrid (Spain) and stored at −20 °C until used. Table 1 shows the composition of the raw material after drying in an oven at 55 °C for 24 h.

### 2.2. HTC and Hydrochar Activation Procedures

HTC was performed in an electrically heated ZipperClave^®^ pressure vessel (4 L), using 1.5 kg of DWAS (15 wt %) in each run. The influence of the process temperature (140–220 °C) and reaction time (0.5–4 h) in the hydrothermal treatment was examined by using the response surfaces provided by a central composite rotatable design. For this purpose, the software Minitab^®^ 19 was used to generate 13 runs (viz., 4 factorial points, 4 axial points and 5 replicates of the central point) with an alpha value of ±1.414. Once the reactor was closed, oxygen was flushed from the system using pure N_2_ (99.99%) for 2 min. The working temperature was reached at a 3 °C/min heating rate. The reaction was stopped by inserting tap water from a serpentine cooler inside the reactor, the reactor was cooled from the desired reaction temperature at a rate lower than 4 °C/min in all the HTC runs. The solid fraction recovered by centrifugation at 3500 rpm for 1 h, washed several times with ethanol and deionized water, and dried at 55 °C for 24 h, the resulting solid was ground and sieved to a particle size of 0.10–0.25 mm. The hydrochar yield (*Y*_HC_), energy densification (*E*_dens_), energy yield (*E*_yield_) and carbon recovery (*C*_recov_) for the process were calculated from the following equations:

(1) $$Y_{HC}\left(\%\right)=\frac{HC\;mass}{DWAS\;mass}\cdot 100.$$

(2) $$E_{dens}=\frac{HHV_{HC}}{HHV_{DWAS}}.$$

(3) $$E_{yield}\left(\%\right)=Y_{HC}\cdot E_{dens}.$$

(4) $$C_{recov}\left(\%\right)=\frac{C_{HC}\cdot HC\;mass}{C_{DWAS}\cdot DWAS\;mass}\cdot 100.$$

The hydrochars were air-activated in a horizontal tube furnace (Nabertherm RHTH 120/300/18/C42) at 300–450 °C for 2 h, using a heating rate of 10 °C/min and an air flow rate of 30 NmL/min [43]. For chemical activation, the hydrochars were mixed with each agent (K_2_CO_3_, KOH, FeCl_3_ or ZnCl_2_, all from Panreac) in a mass ratio of 1:1 at room temperature [43]. This was followed by heating in the previous tube furnace at 650 or 850 °C for 1 h, using a heating rate of 10 °C/min and an N_2_ flow rate of 100 NmL/min. The chemically activated carbons were washed with 1 M HCl and rinsed in an abundance of deionized water to neutral pH [43].

### 2.3. Characterization of Hydrochars and Activated Carbons

The elemental composition (C, N, S, and H) of each material was determined by using a CHNS analyzer (LECO CHNS-932). Moisture, ash and volatile matter (VM) were determined by using ASTM methods D3173-11, D3174-11 and D3175-11, respectively, and higher heating values (HHV) by using a calorimetric bomb (IKA C2000), according to technical specification UNE-EN 5400. Each analysis was performed in triplicate; the standard deviation was less than 5% in all cases. The porous structure of the carbonaceous materials and activated carbons was examined by N_2_ adsorption–desorption at −196 °C in a Micromeritics TriStar II 3020 instrument. Samples were previously outgassed at 100 °C and a residual pressure of 10^−3^ Torr for 8 h. Surface areas (*S*_BET_) were determined from the BET equation [44]. In addition, the surface area and micropore volume of each sample was determined by CO_2_ adsorption at 273 K, and was calculated from the Dubinin–Astakhov equation. The SEM images of samples previously fixed and sputter-coated with gold and were obtained with a Hitachi S-3000N microscope. Fourier-transform infrared (FTIR) spectra were recorded with a BRUKER IFS 66v/S spectrometer. For this purpose, dry samples were mixed with KBr and pressed into pellets that were scanned over the wavenumber region 4000–400 cm^−1^ (250 scans per sample). The metal content of each sample was determined by inductively coupled plasma atomic emission spectroscopy (ICP-MS) on a model Elan 6000 Sciex Perkin Elmer instrument. The slurry pH was determined by using a Crison pH-meter to measure the pH of an aqueous suspension of sample (1 g) in deionized water (10 mL) that had previously been kept under stirring overnight [45].

### 2.4. Adsorption Tests

The potential of the chemically activated carbons (AC) in the aqueous phase adsorbents was assessed by using sulfamethoxazole (SMX), antipyrine (APN) and desipramine (DPN) and were purchased from Sigma–Aldrich as model compounds. The p*K*_a_ values for SMX, APN and DPN were 1.7/5.6, 1.4, and 10.3, respectively [46,47,48]. Samples of AC (12.5 mg) were added to stoppered glass bottles containing 50 mL of aqueous solutions of SMX (25–175 mg/L), APN (25–400 mg/L) and DPN (25–150 mg/L). Tests were carried out in a thermostated shaker (Optic Ivuymen System) at 20 °C at 200 rpm for 120 h, which was long enough for equilibration. SMX, APN and DPN concentrations were determined by UV–Vis spectrophotometry at 265, 246 and 211 nm, respectively, on a Cary 60 UV-Vis instrument from Agilent Technologies. Each result shown is the average of three measurements. The standard error was always less than 5%. Equilibrium data were fitted to the Langmuir equation and all the parameters were calculated by using the non-linear regression fitting method in the software Origin v. 8.5.

## 3. Results and Discussion

### 3.1. Chemical and Structural Properties of the Hydrochars

Table 2 summarizes the physicochemical properties of hydrochars obtained at different temperatures and reaction times. Temperatures below 180 °C resulted in high solid yields but little carbonization. The ash content of the carbonized materials (15–23%) increased with the increasing temperature as a result of the amount of volatile matter (VM) increasing during the HTC process [49,50]. The contents were slightly lower than those reported by Danso-Boateng et al. [10] for hydrochars from sewage sludge (23–39%), and also than those obtained by Kim et al. [49] from the HTC of digested sewage sludge (33%). This was the result of the feedstock used here (DWAS) containing more ash. A response surface methodology was used to examine the influence of temperature (*T*) and reaction time (*t*) on the higher heating value (HHV), yield, and carbon, nitrogen and ash contents, of the hydrochars (Table 3). The equation was obtained using the software Minitab^®^ 19 to generate a central composite rotatable design with 13 runs and an alpha value of ± 1.414. The variables *T*, *T*^2^ and *t*, and the model equations generated were statistically significant (*p* ≤ 0.001) at the 95% confidence level. Both *T* and *t* influenced HHV and the ash content, but only *T* influenced the hydrochar yield, and the carbon and nitrogen contents. This result confirms the critical role of temperature in the HTC process, as observed in previous studies [51].

Table 4 illustrates the energy related properties of the carbon materials. As can be seen, HHV ranged from 19.1 to 22.3 MJ/kg and increased with increasing temperature. Thus, it was 1.27 times greater for the hydrochar carbonized at the highest temperature than it was for the starting feedstock (17.6 MJ/kg). Further, HHV for hydrochar was slightly higher than those for lignite and brown coal (<17.4 MJ/kg) and similar to that for sub-bituminous coal (17–24 MJ/kg). An increased reaction temperature and/or time increased the energy density by enhancing decarboxylation and dehydration reactions [10]. The energy yield exhibited the opposite trend and decreased from 67% to 40%, possibly as a result of the loss of carbon and volatile matter from the hydrochar. 

**Figure 1:**
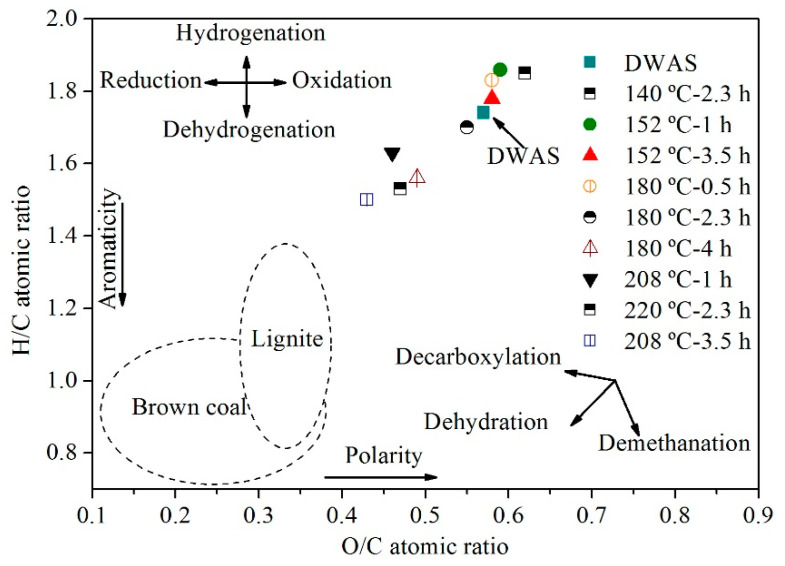
Van Krevelen diagram of DWAS and of carbon materials obtained at different temperatures and reaction times.

The elemental composition of the hydrochars was arranged in the van Krevelen diagram of Figure 1. As can be seen, the H/C and O/C atomic ratios decreased as the reaction temperature was increased from 180 to 220 °C. The fact that HTC reduced the ratios of the starting DWAS confirms the prominent role of dehydration and decarboxylation reactions in addition to hydrolysis [51]. Thus, the H/C and O/C ratios for DWAS were 1.74 and 0.57, respectively, which are rather different from those for brown coal and lignite. As noted earlier, temperatures below 180 °C resulted in insubstantial carbonization. The H/C and O/C ratios for the most carbonized hydrochar (viz., that obtained by HTC at 208 °C for 3.5 h) were 14 and 24% lower, respectively, than those for DWAS.

The content in fixed carbon (FC), which ranged from 8.1 to 15.8% (Table 2), increased with increasing reaction temperature. The hydrochars complied with the quality standard for graded thermally treated and densified biomass fuels for industrial use (ISO 17225-8) [52] in terms of HHV (>17 MJ/kg), sulfur content (<0.5%) and volatile matter content (<75%). However, they exceeded the maximum nitrogen content (<3%) required for reduced NO*_x_* emissions. In any case, this shortcoming can be circumvented by blending the hydrochars with coal or biomass residues [32,38,39]—alternatively, the fuel properties of the hydrochars can be modified with an acid-based treatment [53] to reduce potentially harmful emissions.

In line with previous results [32,41,54,55], the hydrochars exhibited low porosity (*S*_BET_ < 24 m^2^/g). Further insight into their microporosity was gained from the CO_2_ adsorption isotherms at 273 K for the hydrochar obtained by carbonization at 208 °C for 1 h. Thus, the isotherms allowed for a surface area of 252 m^2^/g and a micropore volume of 0.154 cm^3^/g to be calculated. Titirici [56] reported surface areas in the same range for glucose (183 m^2^/g) and sucrose (173 m^2^/g) hydrothermally treated at 180 °C and additionally detected ultramicropores ca. 0.5 µm in size.

**Figure 2:**
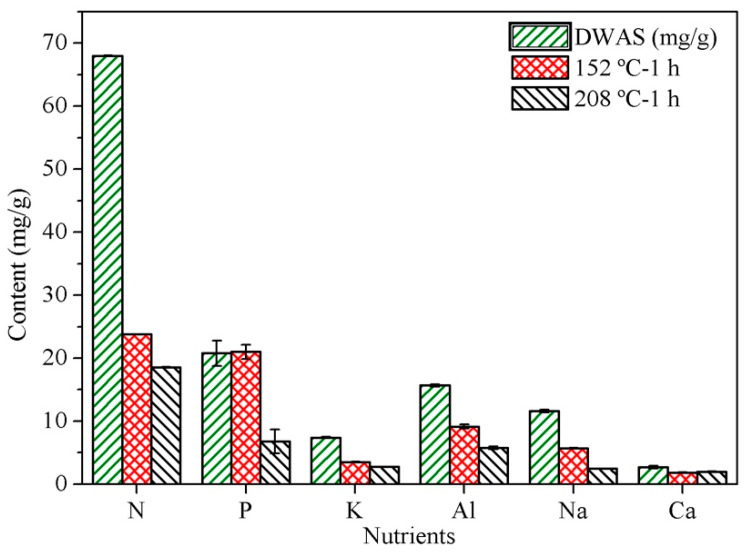
Contents in major nutrients and micronutrients of the carbons materials obtained by heating at 152 and 208 °C for 1 h.

Sewage sludge contains substantial amounts of nutrients such as phosphorus, nitrogen and potassium. These elements are essential nutrients and critical components of most fertilizers. This led us to examine the effect of the process temperature on the persistence of the main macro and micronutrients in the carbon materials. As can be seen from Figure 2, the N, K, and Na contents decreased—and those of the PW increased—as the temperature was raised. Phosphorus was largely retained in the solid materials obtained at low temperatures and only transferred to PW above 152 °C. There are no quality standards for the use of hydrochars to ameliorate soil [57]. Pyrolytic biochars must comply with the requirements of the European Biochar Certificate, which was used as a reference for the hydrochars obtained here [58,59]. Our hydrochars fulfilled the conditions for use as soil ameliorants, in regards to carbon content (>50%) and O/C atomic ratio (<0.4). The macronutrients N, P and K are known to promote plant growth. The NPK proportions of the hydrochars were lower than those of commercial fertilizers (15/15/15) [60]. In particular, they were 6.8/6.0/1.0 and 6.8/2.5/1.0 for the materials obtained by HTC at 152 and 208 °C, respectively, for 1 h. Based on the foregoing, the hydrochars can be used as supplements to improve soil quality while reducing the costs associated to conventional fertilizers. However, some studies have suggested an adverse effect of hydrochars on plant growth [61,62,63,64]. In any case, cleaning hydrochars with water can make them suitable for soil reclamation and agriculture, albeit at an increased cost [65].

### 3.2. Air Activation of Hydrochars

**Figure 3:**
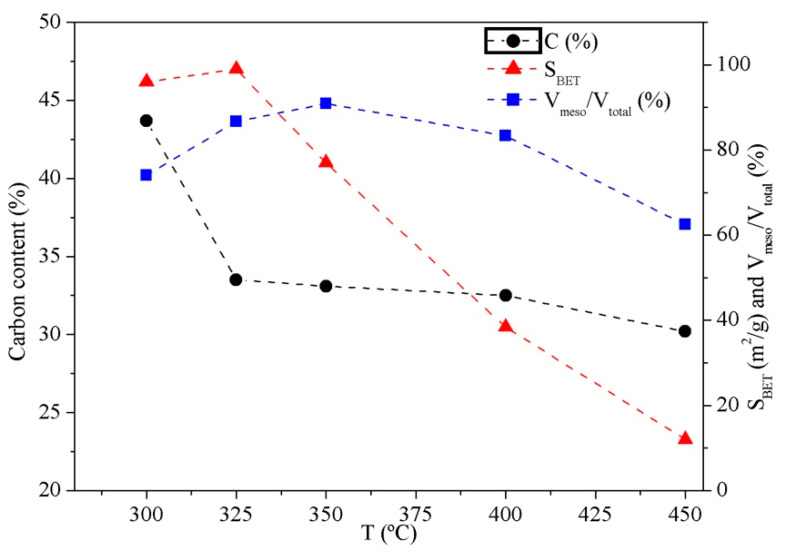
Carbon content on an ash-free basis, surface area (*S*_BET_), and *V*_meso_/*V*_total_ ratio of the hydrochar obtained by heating at 208 °C for 1 h and air-activated at different temperatures.

Mild thermal treatments may have a beneficial effect on the surface functionality (viz., the concentrations of carboxyl, hydroxyl and phenol groups) of low-cost adsorbents [66]. Figure 3 shows the results of activating the hydrochar obtained by carbonization at 208 °C for 1 h by heating at 300–450 °C in the air for 2 h. As can be seen, the BET area of the activated hydrochar decreased as the temperature increased, probably through excessive carbon burn-off, which boosted pore collapse [67,68]. The volume ratio of mesopores to total pores (*V*_meso_/*V*_total_) followed a similar trend and peaked at 0.9 at 350 °C. The carbon content on an ash-free basis decreased from 44% to 30%, and the ash increased content from 61% to 87%, as the temperature was raised (results not shown). Air-activation of the starting DWAS provided a carbonaceous material with a negligible surface area (*S*_BET_ < 3 m^2^/g). This result attests to the importance of an HTC treatment prior to activation for more accurate control of the pore structure.

**Figure 4:**
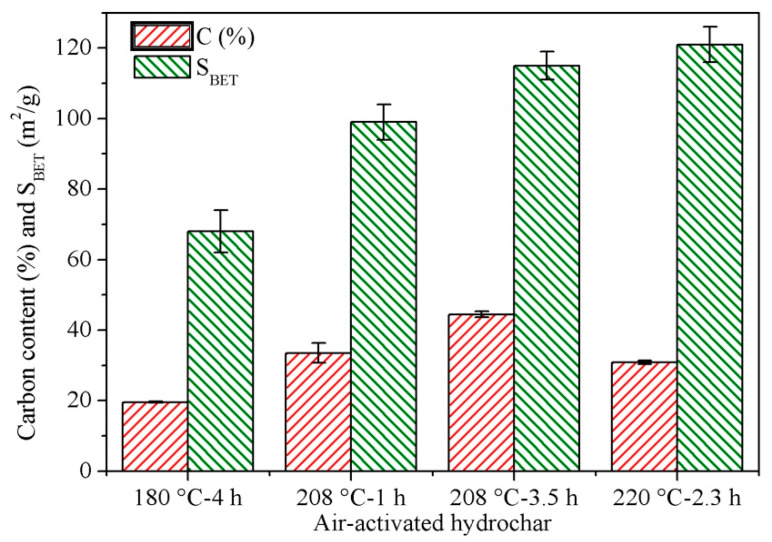
Carbon content on an ash-free basis and surface area (*S*_BET_) of hydrochars air-activated at 325 °C for 2 h.

Well-carbonized hydrochars obtained by HTC at 180 °C for 4 h, 208 °C for 1 or 3.5 h and 220 °C for 2.3 h and were air-activated at 325 °C for 2 h to assess the influence of the carbonization conditions on the pore structure of the resulting carbons. Figure 4 shows the carbon content and surface area of the air-activated hydrochars. As can be seen, air-activation under the stronger conditions increased *S*_BET_. The HTC conditions (temperature, mainly) therefore played a crucial role in the development of the pore network in air-activated carbon materials, which confirms that the surface area and porosity of the resulting hydrochars can be controlled by adjusting the HTC conditions [69].

### 3.3. Chemical Activation of Hydrochars

The hydrochar obtained by heating at 208 °C for 1 h was activated chemically with K_2_CO_3_, KOH, FeCl_3_ and ZnCl_2_ in a single-step process at 650 or 850 °C for 1 h. Table 5 summarizes the properties of the resulting carbons. As can be seen, their surface area increased with increasing temperature and was relatively high for the carbons obtained with KOH (968 m^2^/g), K_2_CO_3_ (832 m^2^/g) and ZnCl_2_ (1030 m^2^/g) at 850 °C, especially if one considers the high ash content of the precursor hydrochar (19.7 wt %). These *S*_BET_ values are similar to those reported by Benstoem et al. [70]. The carbons obtained by activation with FeCl_3_, however, had relatively low surface areas (411–443 m^2^/g), possibly as a result of the high ash content of the material facilitating the incorporation of Fe into the carbonaceous structure. The micropore and mesopore contents of the chemically activated carbons (0.136–0.398 and 0.079–0.271 cm^3^/g, respectively) were both higher than those of the precursor hydrochar (*V*_meso_ = 0.026 cm^3^/g).

### 3.4. Surface Chemistry of the Hydrochars

Figure S1 shows SEM images of DWAS, hydrochars and air-activated hydrochars. As can be seen from Figure S1a,b, DWAS was virtually a nonporous solid of quasi-spherical morphology. However, the hydrochar obtained by HTC at 208 °C for 1 h (Figure S1c,d) was irregularly shaped and contained few pores—probably as a result of recondensation of volatile substances. The hydrochars additionally contained agglomerated and aggregated structures not present in DWAS. As can be seen from Figure S1e,f, the air-activated hydrochars exhibited major changes in their surface, which was rougher and consisted of aggregated microgranules.

Figure S2 shows SEM images of hydrochars activated chemically at 850 °C for 1 h. These materials, with rigid surfaces and well-developed structures, were markedly different from the untreated and air-activated hydrochars. Further, their morphology was strongly dependent on the activation method used. Thus, the surface of the chemically activated hydrochars K_2_CO_3_-AC (Figure S2a,b) and KOH-AC (Figure S2c,d) consisted of irregularly shaped particles, the surface of the former carbon being rougher than that of the latter. By contrast, the carbon FeCl_3_-AC exhibited a heterogeneous morphology and poorly developed porosity (Figure S2e,f). Finally, activation with ZnCl_2_ caused the formation of visible, more prominent cracks with large cavities (Figure S2g,h).

Figure S3 shows the FTIR spectra for DWAS, hydrochars and various physically and chemically activated hydrochars while their FTIR absorption bands are given in Table S1. The main peaks for the carbon obtained by heating at 208 °C for 1 h (Figure S3a) fell in four different spectral regions. The peak associated to hydroxyl groups in the first region was smaller for hydrochars compared to DWAS, which is suggestive of dehydration [71]. Further, the bands associated to asymmetric and symmetric C–H bond stretching of methylene groups at 2970 and 2860 cm^−1^, respectively, in the second region [49] were weaker in the hydrochars, probably as a result of changes in the nonpolar alkyl carbon groups [71]. The peak associated to stretching of the N–O bond in the third region [49] was also smaller, probably because the HTC removed some nitrogen from the DWAS. Finally, the peak assigned to stretching of C–O bonds in alcohols and C–O–R groups in aliphatic ethers [72] in the fourth region increased with increasing temperature, which suggests the dehydration of alcohols [73].

Figure S3b shows the FTIR spectra for air-activated hydrochars obtained at different temperatures. As can be seen, the peaks associated to O–H stretching vibrations in carboxyl or hydroxyl groups decreased with the increasing activation temperature. The band associated to C=N stretching was especially prominent for the carbon obtained at 325 °C. Further, the peaks for C–O and C–O–R in alcohols, phenols, carboxylic acids and esters [74] increased with increasing temperature. Carboxyl, anhydride, lactone and phenolic hydroxyl groups are known to influence the surface acidity of activated carbons. Additionally, the presence of surface acid groups creates their surface polar, which can increase their adsorption capacity for polar alkaline adsorbates such as ammonia, alcohol vapors and water vapor [75,76].

Figure S3c shows the FTIR spectra for chemically activated hydrochars. As can be seen, the band associated with the stretching of O–H bonds, C–O bonds in alcohols and C–O–R bonds in aliphatic ethers in K_2_CO_3_-AC was the strongest. The activated carbons obtained from the hydrochars contained increased amounts of oxygenated (carboxyl, carbonyl and phenol) functional groups.

### 3.5. Adsorption of Sulfamethoxazole, Antipyrine and Desipramine.

**Figure 5:**
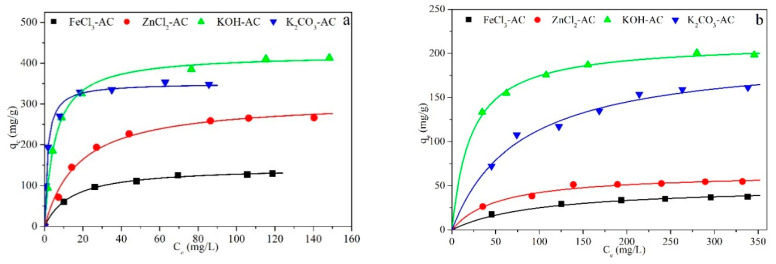

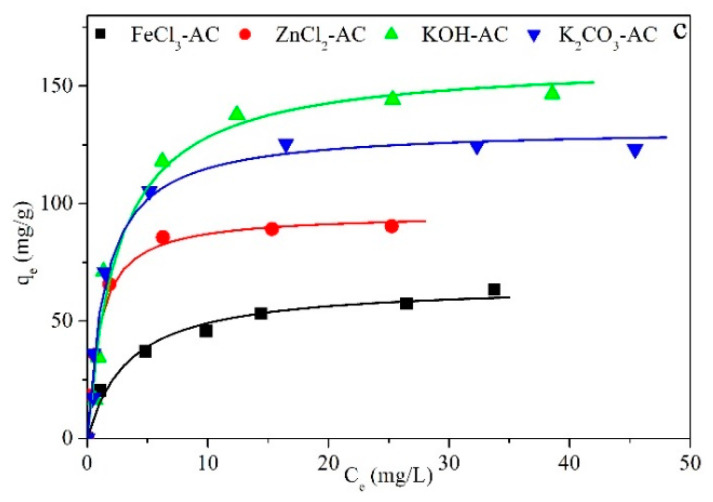
Adsorption isotherms at 20 °C for sulfamethoxazole (**a**), antipyrine (**b**), and desipramine (**c**) on the chemically activated carbons FeCl_3_-AC, ZnCl_2_-AC, KOH-AC and K2CO_3_-AC. Symbols: experimental values. Lines: fitting to the Langmuir equation.

Figure 5 shows the adsorption isotherms at 20 °C for sulfamethoxazole (a), antipyrine (b), and desipramine (c) in the activated carbons obtained by chemical activation at 850 °C. The isotherms are of the L type in the Giles classification [77]. The experimental data were fitted to the Langmuir Equation (10):

(10) $$q_{e}=\frac{q_{L}\cdot K_{L}\cdot C_{e}}{1+K_{L}\cdot C_{e}}$$

where *q_e_* is the equilibrium adsorbate loading onto the adsorbent (mg/g), *C_e_* the equilibrium liquid-phase concentration of adsorbate (mg/L), *q_L_* the monolayer adsorption capacity of the carbon (mg/g) and *K_L_* the Langmuir constant (L/mg).

Table 6 shows the parameter values and correlation coefficients obtained. As can be seen, the results fitted the Langmuir equation quite well. The highest calculated monolayer Langmuir adsorption capacities for SMX, APN and DPN of KOH-AC were 422, 212 and 160 mg/g, respectively, all of which are fairly similar to the experimental values (412, 198 and 146 mg/g, respectively). The adsorption capacity of the activated hydrochars was seemingly influenced more markedly by their mesoporosity than by their BET area, which is consistent with their considerably higher mesopore volumes (KOH ≌ K_2_CO_3_ > ZnCl_2_ > FeCl_3_). The substantial differences in adsorption capacity can be ascribed in part to the low surface area of FeCl_3_-AC relative to the other carbons.

## 4. Conclusions and Future Outlook

Hydrothermal carbonization can be an effective choice for valorizing dewatered waste activated sludge as it provides a renewable solid fuel (hydrochar) with a high energy density and a higher heating value that is similar to that of sub-bituminous coal. As the hydrochar has high ash and nitrogen contents, it should be blended with other biomass waste for combustion if it is to comply with the quality standard of graded thermally treated and densified biomass fuels for industrial use.

Another relevant alternative might be to use the hydrochar as a precursor of activated carbon. Air-activation provides a mild, cost-effective treatment for producing activated carbons with a relatively high surface area (*S*_BET_ = 120 m^2^/g) from DWAS hydrochar. By contrast, chemical activation provides activated carbons with a relatively large surface area (402–1030 m^2^/g), a fairly different porous structure and the potential for use as adsorbents. Mesoporosity in the carbons proved more influential than surface area on their adsorption capacity, the carbons activated with KOH and K_2_CO_3_ exhibiting the highest capacity for the three emerging pollutants studied.

From a technical and economical point of view, further investigation into the design and simulation of the hydrothermal carbonization process is strongly recommended. Very few studies have been focused on determining the energy efficiency of the HTC process through a modeling approach and especially concerning energy integrations and heat recovery. A greater focus on techno-economic analysis could produce interesting findings concern investment and operational costs saving for a large-scale HTC plant coupled with activation processes.

## Figures and Tables

**Table 1:** Composition on a dry basis of the dewatered waste activated sludge.

C (%)	41.5 (0.1)	Na (mg/g)	11.6 (0.2)
H (%)	6.0 (0.1)	Mg (mg/g)	0.7 (0.1)
N (%)	6.8 (0.1)	Al (mg/g)	15.7 (0.2)
S (%)	0.7 (0.1)	P (mg/g)	20.8 (0.4)
O (%) ^a^	31.3 (0.1)	K (mg/g)	7.4 (0.1)
Ash content (%)	13.7 (0.1)	Ca (mg/g)	2.7 (0.2)
Volatile matter (%)	73.6 (0.1)	Ti (mg/g)	0.6 (0.1)
Fixed carbon (%)	12.7 (0.1)	Fe (mg/g)	0.2 (0.1)

^a^ Calculated by difference.

**Table 2:** Composition on a dry basis of the carbons.

Experimental Conditions	Hydrochar Yield (%)	Fixed Carbon (%)	Ash (%)	Volatile Matter (%)	C (%)	H (%)	S (%)	N (%)	O (%) ^a^	C Recovery (%)	*S*_BET_ (m^2^/g)	*V*_meso_^b^ (cm^3^/g)
140 °C—2.3 h	59.7	12.0 (0.1)	15.8 (0.1)	72.5 (0.3)	39.6 (0.3)	6.1 (0.1)	0.3 (0.1)	5.6 (0.1)	32.9 (0.2)	57.0	<3	0.001
152 °C—1 h	61.5	11.5 (0.1)	15.0 (0.1)	73.5 (0.1)	40.6 (0.1)	6.3 (0.1)	0.4 (0.1)	5.9 (0.1)	31.7 (0.1)	60.2	<3	0.001
152 °C—3.5 h	58.5	11.1 (0.1)	17.1 (0.1)	71.8 (3.3)	40.4 (0.9)	6.0 (0.1)	0.2 (0.1)	5.2 (0.1)	31.3 (0.1)	56.9	5	0.006
180 °C—0.5 h	48.5	8.1 (0.1)	16.4 (0.1)	75.4 (1.7)	40.5 (0.1)	6.2 (0.1)	0.3 (0.1)	5.6 (0.1)	31.1 (0.2)	47.3	<3	0.001
180 °C—2.3 h	49.0	13.6 (0.4)	19.3 (0.5)	67.2 (0.5)	40.7 (0.8)	5.8 (0.1)	0.2 (0.1)	4.6 (0.1)	29.5 (0.2)	48.1	15	0.020
180 °C—4 h	46.2	15.5 (0.2)	18.7 (0.1)	65.8 (0.1)	42.7 (0.1)	5.6 (01)	0.2 (0.1)	5.0 (0.1)	27.7 (0.2)	47.5	20	0.027
208 °C—1 h	40.3	14.9 (0.1)	19.7 (0.1)	65.4 (0.3)	43.1 (0.2)	5.8 (0.1)	0.2 (0.1)	4.6 (0.1)	26.5 (0.3)	41.9	21	0.026
208 °C—3.5 h	37.7	15.4 (0.1)	21.3 (0.1)	63.2 (0.1)	43.6 (0.1)	5.5 (0.1)	0.3 (0.1)	4.5 (0.1)	24.9 (0.1)	39.6	23	0.032
220 °C—2.3 h	31.6	15.8 (0.2)	22.8 (0.1)	63.3 (1.6)	41.5 (0.1)	5.3 (0.1)	0.2 (0.1)	4.1 (0.1)	26.1 (0.2)	31.6	24	0.031

^a^ Calculated by difference. ^b^ Volume of mesopores.

**Table 3:** Equations derived from the analysis of variance (ANOVA).

Equation	*R*^2^ (%)	*F*-Value	Number
HHV (MJ/kg) = 13.256 + 0.03662·*T* + 0.361·*t*	0.882	37.2	(5)
Ash content (wt.%) = 0.09392·*T* + 0.782·*t*	0.998	3386.2	(6)
Hydrochar yield (wt.%) = 0.9157·*T* − 0.00354·*T*^2^	0.997	2246.2	(7)
C content (wt.%) = 0.4217·*T* − 0.00106·*T*^2^	0.999	7420.8	(8)
N content (wt.%) = 0.07253·*T* − 0.00025·*T*^2^	0.994	929.4	(9)
*T* temperature (°C). *t* reaction time (h)			

**Table 4:** Energy-related properties of the hydrochars.

Preparation Conditions	HHV (MJ/kg)	Energy Density	Energy Yield (%)
140 °C—2.3 h	19.3 (0.1)	1.10	65.5
152 °C—1 h	19.1 (0.1)	1.09	66.7
152 °C—3.5 h	19.9 (0.1)	1.13	66.1
180 °C—0.5 h	19.5 (0.1)	1.11	53.7
180 °C—2.3 h	20.8 (0.2)	1.18	57.9
180 °C—4 h	21.6 (0.1)	1.23	56.7
208 °C—1 h	21.6 (0.1)	1.23	49.5
208 °C—3.5 h	21.4 (0.5)	1.22	45.8
220 °C—2.3 h	22.3 (0.1)	1.27	40.0

**Table 5:** Selected properties of the chemically activated carbons.

Material	Slurry pH	*T* (°C)	Ash (%)	Fixed Carbon (%)	Elemental Composition (%) ^a^			*S*_BET_ (m^2^/g)	*V*_micro_^b^ (cm^3^/g)	*V*_meso_^c^ (cm^3^/g)
					C	N	S			
K_2_CO_3_-AC	5.5	650	13.1 (0.5)	55.0 (1.3)	61.1 (0.6)	5.8 (0.3)	0.2 (0.1)	583	0.235	0.189
		850	43.4 (0.8)	28.9 (1.0)	34.9 (1.0)	0.5 (0.1)	0.1 (0.1)	832	0.290	0.268
KOH-AC	6.6	650	18.0 (0.5)	52.0 (1.4)	60.9 (2.7)	7.4 (0.2)	0.7 (0.1)	402	0.162	0.079
		850	10.9 (0.4)	67.2 (1.7)	81.0 (3.8)	1.3 (0.1)	0.1 (0.1)	968	0.354	0.271
FeCl_3_-AC	5.1	650	37.6 (0.7)	23.8 (0.5)	39.9 (0.1)	4.8 (0.2)	0.1 (0.1)	443	0.179	0.098
		850	58.4 (1.2)	7.5 (0.3)	28.9 (0.2)	1.8 (0.1)	0.2 (0.1)	411	0.136	0.146
ZnCl_2_-AC	5.7	650	9.4 (0.3)	65.9 (1.3)	66.4 (0.1)	6.9 (0.2)	0.3 (0.1)	661	0.249	0.145
		850	18.6 (0.6)	50.1 (1.6)	57.6 (3.0)	5.3 (0.2)	0.6 (0.1)	1030	0.398	0.204

^a^ Composition data in wt % o.d.b. ^b^ Volume of micropores. ^c^ Volume of mesopores.

**Table 6:** Parameters of the Langmuir equation for the adsorption of sulfamethoxazole, antipyrine and desipramine on chemically activated carbons obtained at 850 °C.

**Sulfamethoxazole**				
**Parameter**	**FeCl_3_-AC**	**ZnCl_2_-AC**	**KOH-AC**	**K_2_CO_3_-AC**
***q*_L_ (mg/g)**	145.8 (2.3)	309.2 (9.6)	422.9 (3.9)	350.5 (7.1)
***K*_L_ (L/mg)**	0.07 (0.01)	0.06 (0.01)	0.19 (0.01)	0.68 (0.09)
***R*^2^**	0.997	0.991	0.998	0.991
**Antipyrine**				
**Parameter**	**FeCl_3_-AC**	**ZnCl_2_-AC**	**KOH-AC**	**K_2_CO_3_-AC**
***q*_L_ (mg/g)**	50.0 (2.4)	64.5 (2.6)	212.6 (2.2)	200.7 (8.7)
***K*_L_ (L/mg)**	0.01 (<0.01)	0.02 (<0.01)	0.05 (<0.01)	0.01 (<0.01)
***R*^2^**	0.992	0.986	0.998	0.989
**Desipramine**				
**Parameter**	**FeCl_3_-AC**	**ZnCl_2_-AC**	**KOH-AC**	**K_2_CO_3_-AC**
***q*_L_ (mg/g)**	65.9 (3.1)	95.8 (2.0)	160.7 (8.5)	132.1 (4.4)
***K*_L_ (L/mg)**	0.29 (0.06)	1.00 (0.10)	0.40 (0.09)	0.67 (0.11)
***R*^2^**	0.982	0.995	0.971	0.983
Errors represent the 95% confidence interval.				

## Supplementary Materials

The following are available online at https://www.mdpi.com/1420-3049/25/15/3534/s1, Figure S1: SEM images of DWAS (a, b), hydrochar (208 °C for 1 h) (c, d), and air-activated hydrochar (325 °C for 2 h) (e, f). Figure S2: SEM images of K_2_CO_3_-AC (a, b), KOH-AC (c, d), FeCl_3_-AC (e, f), and ZnCl_2_-AC (g, h) activated at 850 °C for 1 h. Figure S3: FTIR spectra of dewatered waste activated sludge and carbon materials obtained at different temperatures and reaction times (a); air-activated carbons obtained at several temperatures (b); and chemically-activated carbons obtained at 650 and 850 °C (c). Table S1: Assignment of Fourier transform infrared (FTIR) spectroscopy absorption bands of DWAS and several hydrochars physical and chemically activated.

## Abbreviations

AC: activated carbon; APN: antipyrine; COD: chemical oxygen demand; DPN: desipramine; DWAS: dewatered waste activated sludge; FC: fixed carbon; FTIR: Fourier-transform infrared spectroscopy; HC: hydrochar; HHV: higher heating value; HTC: hydrothermal carbonization; SMX: sulfamethoxazole; VM: volatile matter.
