# Supplementary material for: Potential Use of Waste Activated Sludge Hydrothermally Treated as a Renewable Fuel or Activated Carbon Precursor
Source: Molecules. 2020 Aug 2;25(15):3534. doi: 10.3390/molecules25153534 (PMC7435997; doi:10.3390/molecules25153534)
Supplement: Supplementary file 1 [file molecules-25-03534-s001.pdf]

Supplementary Material

Potential use of waste activated sludge hydrothermally treated as a renewable fuel or activated carbon precursor

J. A. Villamil [1,*], E. Diaz [2], M.A. De la Rubia [2] and A. F. Mohedano [2]

[1] Departamento de Tecnología Química y Ambiental. Universidad Rey Juan Carlos, 28933 Móstoles (Madrid), Spain

[2] Departamento de Ingenieria Química, Facultad de Ciencias, Universidad Autonoma de Madrid, Campus de Cantoblanco, 28049 Madrid, Spain

* Correspondence: john.villamil@urjc.es

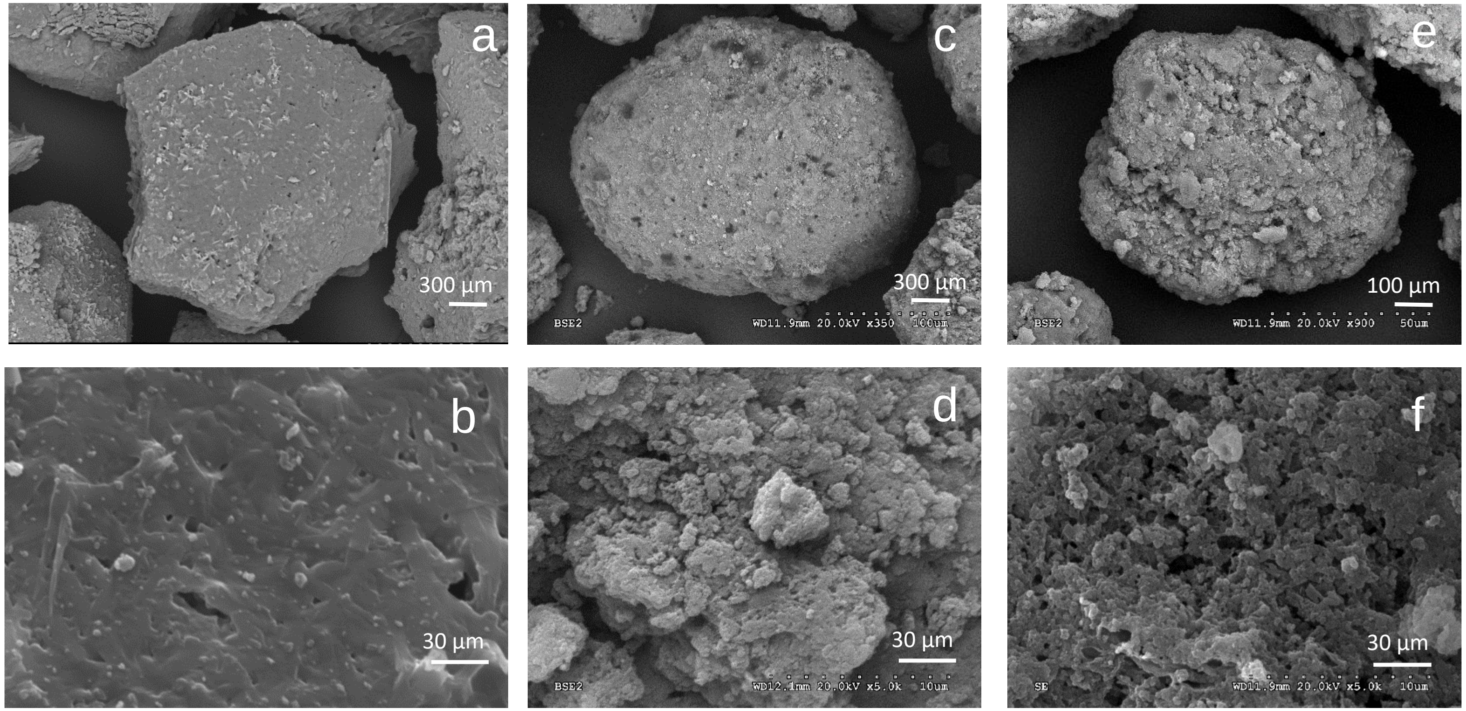

Figure S1. SEM images of DWAS (**a, b**), hydrochar (208 °C for 1 h) (**c, d**), and air-activated hydrochar (325 °C for 2 h) (**e, f**).

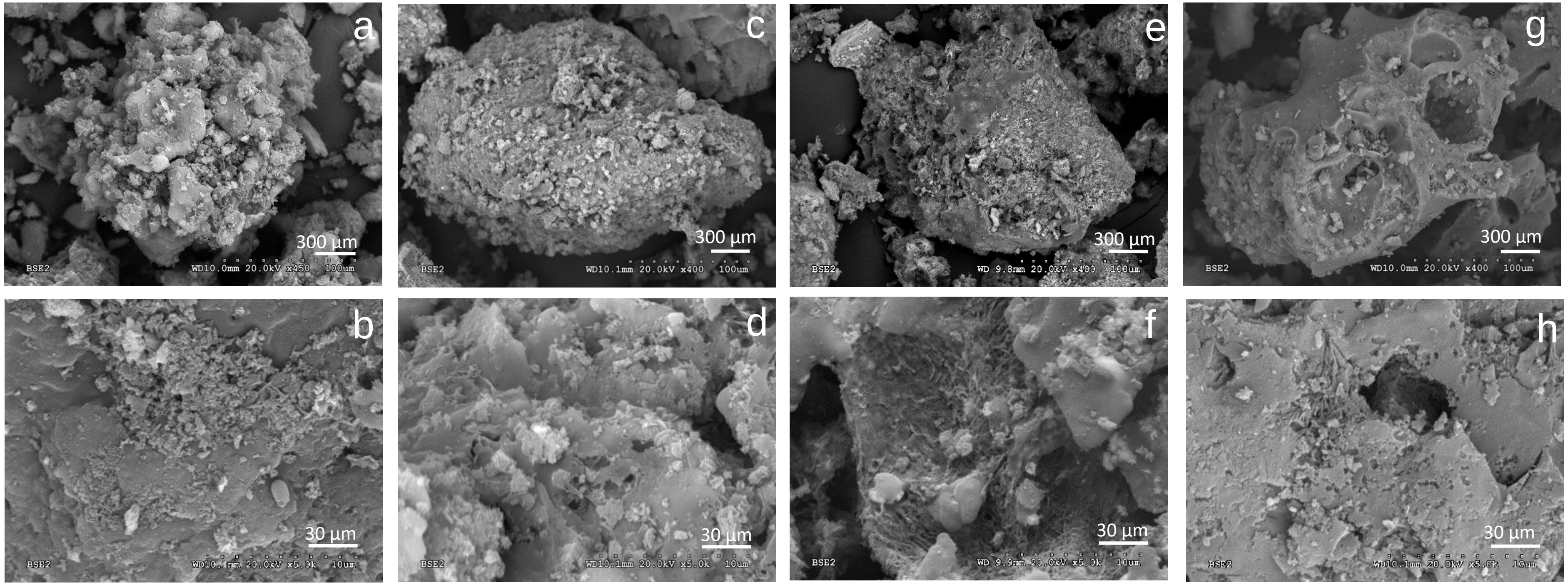

Figure S2. SEM images of K_2CO_3-AC (**a, b**), KOH-AC (**c, d**), $FeCl_3$-AC (**e, f**), and $ZnCl_2$-AC (**g, h**) activated at 850 °C for 1 h.

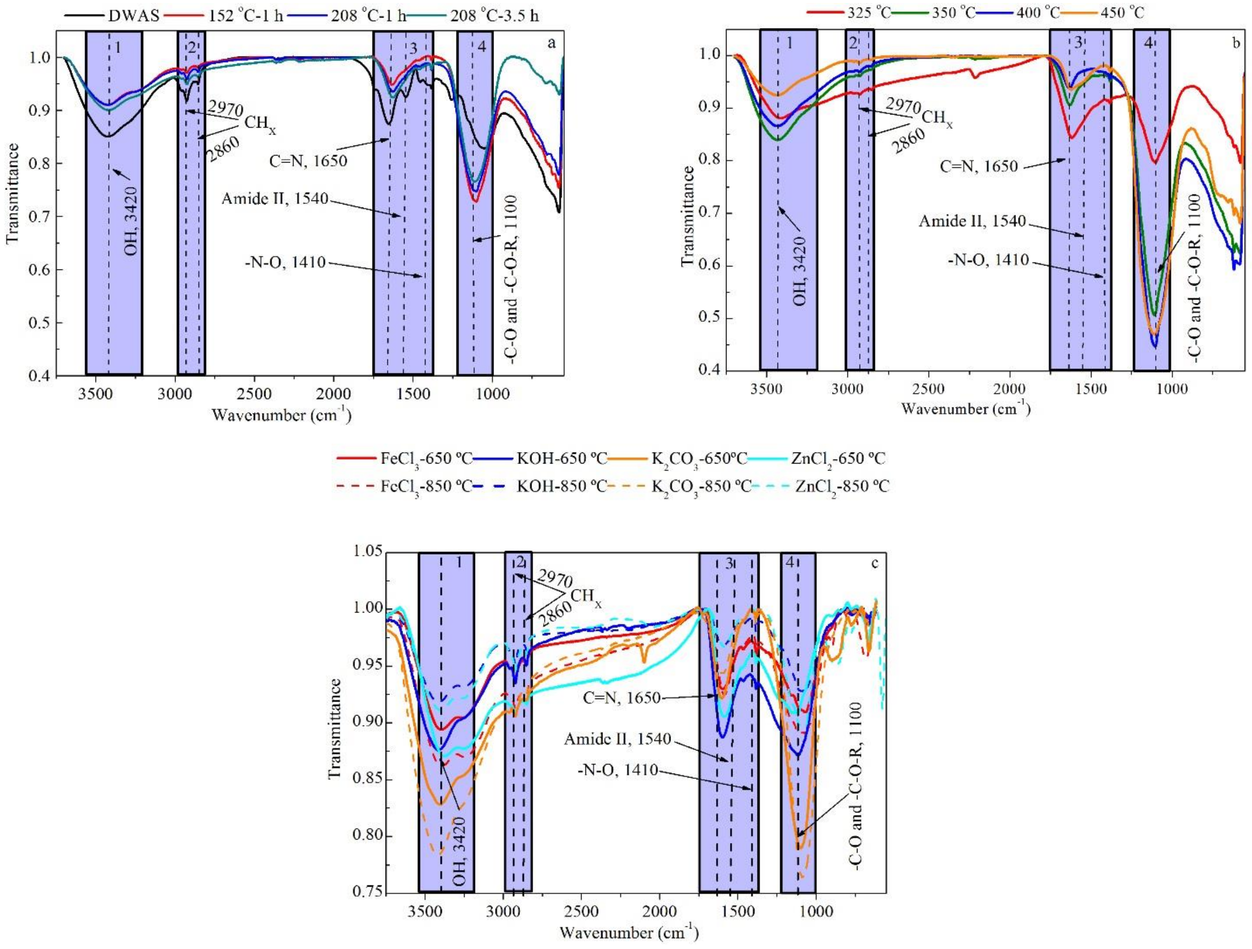

Figure S3. FTIR spectra of dewatered waste activated sludge and carbon materials obtained at different temperatures and reaction times (**a**); air-activated carbons obtained at several temperatures (**b**); and chemically-activated carbons obtained at 650 and 850 °C (**c**).

Table S1. Assignment of Fourier transform infrared (FTIR) spectroscopy absorption bands of DWAS and several hydrochars physical and chemically activated.

Region	σ (cm^{-1})	Assignment	Designation
I (3550–3150 cm^{-1})	3420	υ (OH)	Attributed to -OH stretching vibration in carboxyl or hydroxyl groups
II (3000–2750 cm^{-1})	2970, 2860	υ (C-H)	Attributed to the asymmetric and symmetric –C–H stretching of the methylene groups
	1650	υ (C=N)	Associated with C=N amides
III (1750–1300 cm^{-1})	1540	υ (-N-H)	Attributed to -N-H in-plane bending of amide II and secondary amines
	1410	υ (N-O)	Associated with the N-O group
IV (1200–950 cm^{-1})	1100	υ (-C-O), υ (C-O-R)	Associated with the functional group as alcohols, phenols, carboxylic acids, and esters
